# Repellent and insecticidal efficacy of a new combination of fipronil and permethrin against three mosquito species (*Aedes albopictus, Aedes aegypti* and *Culex pipiens*) on dogs

**DOI:** 10.1186/s13071-015-0691-y

**Published:** 2015-01-30

**Authors:** Becky Fankhauser, Pascal Dumont, James S Hunter, John W McCall, Christian Kaufmann, Alexander Mathis, David R Young, Scott P Carroll, Scott McCall, S Theodore Chester, Mark D Soll

**Affiliations:** Merial Limited, 3239 Satellite Blvd, Duluth, GA 30096 USA; Merial S.A.S., 29 Av Tony Garnier, 69007 Lyon, France; TRS Labs, Inc., 295 Research Drive, Athens, GA 30605 USA; Institute of Parasitology, Vetsuisse Faculty, University of Zurich, Winterthurerstrasse 266a, 8057 Zurich, Switzerland; Young Veterinary Research Services, 7243 East Avenue, Turlock, CA 95380-9124 USA; Carroll-Loye Biological Research, 711 Oak Avenue, Davis, CA 95616 USA

**Keywords:** Mosquitoes, *Aedes aegypti*, *Aedes albopictus*, *Culex pipiens*, Permethrin, Fipronil, Dog, Repellency, Frontline Tri- Act®/Frontect®

## Abstract

**Background:**

Three laboratory studies were conducted to assess the repellent and insecticidal efficacy of a combination of fipronil and permethrin (Frontline Tri- Act®/Frontect®) against three mosquito species (*Aedes albopictus, Aedes aegypti* and *Culex pipiens*) on dogs*.*

**Methods:**

In each study, 16 healthy adult dogs were allocated to two groups. Eight dogs were treated with the new topical spot-on combination of fipronil and permethrin on Day 0 and the other eight dogs served as untreated controls. Each dog was exposed to mosquitoes on Days 1, 7, 14, 21 and 28 (and also on Day 35 in the *A. aegypti* study). After a 1-h exposure period, all mosquitoes were counted and categorized as live or dead and fed or non-fed. Live mosquitoes were kept in an insectary and observed for mortality counts 4, 24 and 48 h post-exposure (PE) for *Aedes* spp. and 24 and 48 h PE for *C. pipiens*. Repellency and insecticidal efficacies were defined as the percent reduction in the number of fed and live mosquitoes, respectively, in the treated group as compared to the untreated control group.

**Results:**

Repellency against *A. albopictus* was ≥93.4% through Day 21 and 86.9% on Day 28. It was ≥91.0% through Day 35 against *A. aegypti* and ≥90.4% through Day 28 against *C. pipiens.* Insecticidal efficacy against *A. albopictus* was ≥97.1% at 24 h PE from Day 7 to Day 28. It was ≥98.0% for the first 3 weeks and still 75.7% on Day 35 against *A. aegypti* at 24 h PE. For *C. pipiens*, insecticidal efficacy ranged from 93.8% (Day 7) to 30.9% (Day 28) at 48 h PE.

**Conclusions:**

A single topical administration of the combination of fipronil and permethrin provides repellency against mosquitoes on dogs for at least 4 weeks. The product may therefore significantly reduce the potential for the transmission of vector-borne pathogens through the inhibition of mosquito feeding, as well as the discomfort associated with mosquito bites. Moreover, mosquito mortality was induced by contact with the treated dogs, which could aid in the control of mosquitoes, and hence the control of mosquito-borne diseases, in the local vicinity of treated dogs.

## Abrégé

**Contexte:**

Trois études expérimentales ont été conduites afin de mesurer l’efficacité répulsive et insecticide d’une combinaison de fipronil et perméthrine (Frontline Tri- Act®/Frontect®) contre trois espèces de moustiques (*Aedes albopictus*, *Aedes aegypti* et *Culex pipiens*) chez le chien.

**Méthodes:**

Les protocoles des études étaient similaires. Dans chaque étude, 16 chiens adultes en bonne santé ont été répartis en deux groupes. Huit chiens ont été traités avec la nouvelle combinaison spot-on topique de fipronil et perméthrine au jour 0 et les huit autres chiens servaient de témoins non traités. Chaque chien a été exposé aux moustiques aux jours 1, 7, 14, 21 et 28 (également au jour 35 dans l’étude *A. aegypti*). Après une période d’une heure d’exposition, tous les moustiques ont été comptés et catégorisés : vivants ou morts, gorgés ou non gorgés. Les moustiques vivants ont été placés en insectarium et leur mortalité observée 4, 24 et 48 h post-exposition (PE) pour *Aedes* spp. et 24 et 48 h PE pour *C. pipiens*. Les efficacités répulsives et insecticides sont respectivement définies comme le pourcentage de réduction du nombre de moustiques gorgés ou vivants dans le groupe traité comparé au groupe témoin non traité.

**Résultats:**

La répulsivité contre *A. albopictus* était ≥ 93,4% jusqu’au jour 21 puis 86,9% au jour 28. Elle était ≥ 91,0% jusqu’au jour 35 pour *A. aegypti* et ≥ 90,4% jusqu’au jour 28 pour *C. pipiens*. L’efficacité insecticide vis-à-vis de *A. albopictus* était ≥ 97,1% 24 h PE du jour 7 au jour 28. Elle était ≥ 98,0% pendant les 3 premières semaines et 75,7% au jour 35 vis-à-vis de *A. aegypti* 24 h PE. Pour *C. pipiens*, l’efficacité insecticide était de 93,8% (jour 7) à 30,9% (jour 28) 48 h PE.

**Conclusions:**

Dans cette étude, une administration topique unique de la combinaison de fipronil et perméthrine a permis une activité répulsive d’au moins 4 semaines contre les moustiques chez le chien. Le produit pourrait de ce fait réduire significativement leur potentiel de transmission d’agents pathogènes vectorisés, par l’inhibition du gorgement des moustiques, mais réduire aussi l’inconfort lié aux piqûres de moustiques. De plus, le contact avec le chien traité a provoqué la mort des moustiques. Ce bénéfice pourrait apporter une aide dans la lutte contre les moustiques dans l’environnement proche du chien traité et dans le contrôle des maladies transmises par les moustiques, menaces émergentes pour la santé humaine et animale.

## Background

Mosquitoes form a monophyletic family of insects, Culicidae, that inhabit practically every region of every continent in the world except Antarctica and are of significant importance in human and veterinary medicine [1]. Mosquito bites can cause pets discomfort and irritation, and can lead to hypersensitivity reactions in some animals. More importantly, mosquitoes are vectors of several pathogens of considerable importance to pets, including helminths such as *Dirofilaria repens* (subcutaneous filarial worm) and *Dirofilaria immitis* (heartworm) as well as several viruses affecting both humans and dogs.

*Dirofilaria repens* infection is typically confined to the subcutaneous tissues in dogs and infected animals may be apparently healthy or may develop skin conditions of varying severity [2]. The infection is zoonotic and can cause an array of clinical signs in humans, from benign to very severe conditions. The number of reported cases has been increasing in recent years. *Culex* and *Aedes* are the main species involved in the transmission of *D. repens* in both animals and humans [3]*.* Heartworm disease is a severe and potentially fatal disease in dogs that is endemic in the United States of America (USA), much of southern Europe, Australia, several countries in South America, and in many countries in Asia [4]. While many species of mosquitoes can transmit heartworms*, Aedes aegypti, Aedes albopictus* and *Culex pipiens* are among the most important vectors in many parts of the world [5-10]. For example, it was recently demonstrated that *C. pipiens* is the most efficient natural vector of *D. immitis* in endemic areas of Italy based on its high feeding rate on dogs and the detection rate of *D. immitis* in sampled mosquitoes [11]. Although less important in pets than in human health, arboviruses may also cause severe disease in dogs. Mosquito-borne viruses of pets are found in three viral families: Alphaviridae, Flaviviridae and Bunyaviridae [12]. Among Alphaviridae, viruses from the complexes responsible for equine encephalitis transmitted by *Aedes* spp. and *Culex* spp. may cause severe neurological signs in dogs. The West Nile virus and Japanese encephalitis virus from the Flaviviridae family are mainly transmitted by *Culex* spp. West Nile virus has spread from Africa to Europe, Asia and North America. Seroprevalence in dogs in endemic areas is high and clinical cases, despite being rare, may be severe. Three members of the Bunyaviridae family are known to cause diseases in dogs: the Lacrosse encephalitis virus transmitted by *Aedes triseriatus* in North America, the Tensaw virus transmitted by *Anopheles* spp. in Southeastern United States and the Rift Valley fever virus transmitted by *Culex* spp. and *Aedes* spp. in Eastern Africa [12].

*Aedes albopictus*, the Asian tiger mosquito, probably presents the most important threat of any mosquito species to public health in Europe. It is considered to be the most invasive mosquito species in the world and, despite the efforts made in controlling its spread, it is now well established in new territories, and in Europe its control is a major concern of authorities [13]. It originated in Southeast Asia but in the last 30 years has spread into Europe, North and South America, Africa, and a number of locations in the Pacific and Indian Oceans [14]. Unlike most mosquito species, *A. albopictus* feed during the day, thus rendering previous prevention strategies such as keeping animals indoors at night ineffective [11]. The rapid spread, aggressive nature, ability to transmit both helminthic and viral pathogens and the diurnal feeding behavior of *A. albopictus* make it a particular concern for pet dogs.

An integrated approach to vector and transmitted pathogen control, consisting of using all available management strategies to limit the disease burden, including the additional application of a parasiticide with repellent (i.e. anti-feeding) effect against mosquitoes, can help provide optimal protection against vector-borne diseases for dogs and humans in endemic areas. In addition, products that can potentially impact the mosquito population in the local vicinity of treated dogs are of interest as well.

Permethrin is a Type 1 pyrethroid, which is an insecticide and acaricide with repellent activity. Permethrin has been applied in different formulations for the control of ectoparasites on both companion and production animals, as well as humans. Both single-active formulations, as well as combination products, have been shown to effectively control mosquitoes [9,15,16]. Fipronil, a phenylpyrazole, has both insecticidal and acaricidal activity. Fipronil has been used in spray and spot-on formulations to control fleas and ticks on companion animals [17-20].

A novel combination of 6.76% w/v fipronil and 50.48% w/v permethrin (Frontline Tri- Act®/Frontect®)) has been developed for use as a monthly topical solution for dogs to provide broad spectrum ectoparasite control. The studies reported here were conducted to assess the repellent and insecticidal efficacies of this combination against three species of mosquitoes (*A. albopictus, A. aegypti* and *C. pipiens).*

## Methods

### Study design

Three studies, each with a similar study design, were conducted. Study 1 used *A. albopictus* mosquitoes, Study 2 used *A. aegypti* mosquitoes, and Study 3 used *C. pipiens quinquefasciatus* mosquitoes (part of the *C. pipiens* complex; these mosquitoes will be referred to simply as *C. pipiens* in this and the other sections of this manuscript). Studies 1 and 2 were conducted in the USA and Study 3 was conducted in Switzerland. All studies were conducted in accordance with Good Clinical Practices (GCP) as described in the International Cooperation on Harmonisation of Technical Requirements for Registration of Veterinary Medicinal Products (VICH) guideline 9.

### Animals

For each study, 18 dogs were exposed to mosquitoes prior to enrollment to assess their susceptibility to mosquito feeding. The two dogs with the lowest number of fed mosquitoes after this exposure were dropped from the study. The remaining 16 dogs were ranked by descending fed mosquito count and eight blocks of two dogs each were formed. Within blocks, each dog was randomly allocated to one of two groups. The enrolled dogs were healthy Beagle dogs with a mixture of males and females in each study (10 males and 6 females in Study 1; 9 males and 7 females in Study 2; 7 males and 9 females in Study 3) and had not been exposed to ectoparasiticides in the 3 months prior to the study. The dogs were managed with due regard for their well-being, in accordance with Merial and local Institutional Animal Care and Use Committee requirements. Dogs were housed individually in Studies 1 and 2, and were housed in groups of 2 or 3 within treatment group in Study 3 (*C. pipiens*) due to local laws restricting individual housing. A veterinary examination was performed prior to the start of each study to ensure that all dogs were healthy and suitable for inclusion in the study. The dogs were observed daily for any health changes throughout each study.

### Treatment

Dogs in Group 1 received no treatment and served as untreated controls. Dogs in Group 2 were treated once with a topical formulation containing 6.76% w/v fipronil and 50.48% w/v permethrin. In Studies 1 and 2 (*A. albopictus* and *A. aegypti*, respectively)*,* the dogs were treated with the commercial dose of the product based on their body weight. In Study 3 (*C. pipiens*), each dog was dosed with the minimum dose of the product (0.1 ml/kg; 6.76 mg/kg fipronil, 50.48 mg/kg permethrin) once topically. In each study, the total volume of the product was divided into two approximately equal fractions and placed on the skin on the midline of the neck. One fraction was applied between the base of the skull and the shoulder blades and the other was applied at the front of the shoulder blades. All dogs were observed hourly for any adverse reaction for 4 h post-treatment.

### Mosquitoes

All mosquitoes were laboratory-reared. Five-to-10-day old female *A. albopictus* were used in Study 1. The *A. albopictus* mosquitoes were sourced as eggs from three laboratory colonies in the USA (Gainesville and Vero Beach, Florida, and Raleigh, North Carolina). Four-to-5-day old female *A. aegypti* (Liverpool black-eyed strain) were used in Study 2. Seven-to-14-day old female *C. pipiens* were used in Study 3. This strain was isolated from California, USA, and has been maintained in Toulouse, France, since 1984, and in Zurich, Switzerland, since 2011.

### Mosquito exposures and counts

Dogs were exposed to mosquitoes on Days 1, 7, 14, 21 and 28 (and also on Day 35 in Study 2). Prior to exposure, each dog was anesthetized using one of the following combinations administered intramuscularly: 11 mg/kg ketamine (Ketaset®, Zoetis) and 2.2 mg/kg xylazine (Xylazine-20, Butler Co.); 0.02 mg/kg dexmedetomidine (Dexdomitor®, Orion Corporation) and 0.2 mg/kg butorphanol (Butorphic®, Lloyd Laboratories); 2.9 mg/kg ketamine (Ketaset®, Zoetis) and 1.1 mg/kg xylazine (AnaSed®,Lloyd Laboratories); or 0.04 mg/kg medetomidine (Dorbene®, Graeub) and 0.2 mg/kg butorphanol (Morphasol-4®, Graeub). Additional amounts of the anesthetics were administered as needed to maintain anesthesia for the duration of the exposure period. Once anesthetized, each dog was placed into an individual mosquito proof exposure cage and mosquitoes (approximately 100 female *A. aegypti* or *C. pipiens,* and approximately 75 female *A. albopictus*) were released into the exposure cage. After the 1-hour exposure period, live mosquitoes were counted and categorized as fed or non-fed and then held in a container in an insectary for subsequent mortality counts. Dead mosquitoes remaining in the cage or on the dog were counted and categorized as fed or non-fed. Any mosquitoes that were physically crushed by the dog were noted but were not used in the assessment of repellency or insecticidal efficacy. At approximately 4, 24 and 48 h post-exposure (PE) for Studies 1 and 2 (*Aedes* spp.) and 24 and 48 h for Study 3 (*C. pipiens*), the number of dead mosquitoes in each holding container was counted and used to determine the number of mosquitoes remaining alive. In Studies 2 and 3, whether live mosquitoes had fed or not was visually determined immediately after the exposure period. In Study 1 (*A. albopictus*), the live mosquitoes were very active and difficult to count. To improve accuracy of the counts of this species, live mosquitoes were frozen after the last mortality count and counted as fed or non-fed after freezing (engorgement status can be evaluated up to 48 h post-blood feeding). All personnel conducting mosquito counts and health observations were blinded to treatment groups.

### Data analysis

Percent mosquito repellency was defined as the percent reduction in the number of fed mosquitoes in the treated group as compared to the untreated control group. The total numbers of fed (live + dead) mosquitoes at the end of each exposure period were transformed to the natural logarithm of (count + 1) for calculation of geometric means (GM) by treatment group. Percent repellency at each post-treatment exposure day was calculated as 100 × [(C – T)/C], where C is the GM of the control group and T is the GM of the treated group. Crushed mosquitoes were not included in the analysis.

Percent insecticidal efficacy was defined as the percent reduction in live mosquitoes in the treated group as compared to the untreated control group. The number of live mosquitoes at each time point (4, 24 and 48 h in Studies 1 and 2, and 24 and 48 h in Study 3) after each post-treatment exposure was transformed to the natural logarithm of (count + 1) for calculation of GM by treatment group at each time point of each post-treatment exposure. Percent insecticidal efficacy of the treated group compared to the control group at each time point of each post-treatment exposure was calculated as 100 x [(C – T)/C], where C is the mean of the control group and T is the mean of the treated group.

For both repellency and insecticidal efficacy, the treated group was compared to the untreated control group using the Friedman rank test with blocks defined as the allocation blocks. The testing was two-sided and used a significance level of 5%. The analyses were performed using SAS® Version 9.1.3.

## Results

### Repellency

There was a significant difference between the population means of the treated and control groups at every time point (*p* = 0.005) for each mosquito species.

The mosquito challenge was evaluated by the number of fed mosquitoes in the untreated control dogs at the end of each exposure period. The GM of fed mosquitoes in the untreated control dogs ranged from 18.3 to 38.0 in Study 1 (75 *A. albopictus* used for each exposure), from 68.2 to 85.3 in Study 2 (100 *A. aegypti* used for each exposure), and from 23.9 to 76.8 in Study 3 (100 *C. pipiens* used for each exposure) (Tables 1, 2 and 3).

**Table 1:** **Percent repellency of** ***Aedes albopictus*** **in dogs treated with a new combination of fipronil and permethrin**

**Exposure day**	**GM of numbers of fed mosquitoes** ^**1**^		**Repellency (%)**
	**Untreated control dogs**	**Treated dogs**	
	**(n = 8)**	**(n = 8)**	
1	18.3	1.2	93.4*
7	20.8	0.7	96.5*
14	28.8	0.1	99.5*
21	38.0	1.3	96.6*
28	23.0	3.0	86.9*

^1^Geometric Means of mosquitoes collected at the end of the 1-h exposure period. 75 mosquitoes per dog were used for each exposure.

*Significant difference between the population means of the treated and control groups (*p* = 0.005).

**Table 2:** **Percent repellency of** ***Aedes aegypti*** **in dogs treated with a new combination of fipronil and permethrin**

**Exposure day**	**GM of numbers of fed mosquitoes** ^**1**^		**Repellency (%)**
	**Untreated control dogs**	**Treated dogs**	
	**(n = 8)**	**(n = 8)**	
1	68.2	0.1	99.9*
7	84.4	0.0	100*
14	83.9	0.8	99.0*
21	80.6	1.2	98.5*
28	83.0	3.3	96.1*
35	85.3	7.7	91.0*

^1^Geometric Means of mosquitoes collected at the end of the 1-h exposure period. 100 mosquitoes per dog were used for each exposure.

*Significant difference between the population means of the treated and control groups (*p* = 0.005).

**Table 3:** **Percent repellency of** ***Culex pipiens*** **in dogs treated with a new combination of fipronil and permethrin**

**Exposure Day**	**GM of numbers of fed mosquitoes** ^**1**^		**Repellency (%)**
	**Untreated control dogs**	**Treated dogs**	
	**(n = 8)**	**(n = 8)**	
1	23.9	0.1	99.4*
7	45.2	0.5	98.9*
14	52.7	2.8	94.7*
21	76.8	6.4	91.7*
28	62.5	6.0	90.4*

^1^Geometric Means of mosquitoes collected at the end of the 1-h exposure period. 100 mosquitoes per dog were used for each exposure.

*Significant difference between the population means of the treated and control groups (*p* = 0.005).

#### Aedes albopictus

The GM number of fed *A. albopictus* in each group and the percent repellency after the 1 h exposure period on each exposure day are shown in Table 1. Repellency was 93.4%, 96.5%, 99.5%, 96.6% and 86.9% on Days 1, 7, 14, 21 and 28, respectively.

#### Aedes aegypti

The GM number of fed *A. aegypti* in each group and the percent repellency after the 1 h exposure period on each exposure day are shown in Table 2. Repellency was 99.9%, 100%, 99.0%, 98.5%, 96.1% and 91.0% on Days 1, 7, 14, 21, 28 and 35, respectively.

#### Culex pipiens

The GM number of fed *C. pipiens* in each group and the percent repellency after the 1 h exposure period on each exposure day are shown in Table 3. Repellency was 99.4%, 98.9%, 94.7%, 91.7%, and 90.4% on Days 1, 7, 14, 21, and 28 respectively.

### Insecticidal efficacy

In each study, high numbers of mosquitoes exposed to the untreated control dogs remained alive at all PE time points after each exposure except on Day 1 for *A. albopictus* (Study 1). In this study, no evaluation was performed on the data collected after the Day 1 exposure because all of the mosquitoes in the control group were dead at the end of the PE holding period (48 h). This was likely due to dehydration of the mosquitoes as the source of water and carbohydrate dried out more quickly than anticipated. In the subsequent exposures, these sources were replenished frequently and no additional problems with mosquito mortality in the control group were noted.

Treated dogs had significantly fewer live mosquitoes than untreated control dogs in all studies and for all study days and time points. The percent insecticidal efficacy for each mosquito species at each time point for each exposure day is shown in Table 4.

**Table 4:** **Percent insecticidal efficacy of** ***Aedes albopictus, Aedes aegypti*** **and** ***Culex pipiens*** **in dogs treated with a new combination of fipronil and permethrin**

**Species**	**Time post exposure** ^**1**^	**Percent insecticidal efficacy** ^**2**^					
		**Day 1**	**Day 7**	**Day 14**	**Day 21**	**Day 28**	**Day 35**
*A. albopictus*	4 h	ND^3^	100*	93.2*	93.7*	91.1*	ND
	24 h	ND^3^	100*	97.1*	100*	100*	ND
	48 h	ND^3^	100*	97.8*	100*	100*	ND
*A. aegypti*	4 h	99.8*	93.8*	69.5*	77.0*	20.9*	27.1*
	24 h	99.7*	99.9*	98.0*	98.5*	79.8*	75.7*
	48 h	99.7*	99.8*	99.1*	99.3*	86.6*	84.7*
*C. pipiens*	24 h	77.9*	92.1*	34.7*	38.5*	26.9*	ND
	48 h	79.4*	93.8*	37.3*	45.0*	30.9*	ND

^1^Mosquitoes were collected at the end of the 1-h exposure period. ^2^Based on geometric means.

^3^Analysis not performed on these days.

*Significant difference between the population means of the treated and control groups (*p* = 0.005).

#### Aedes albopictus

The insecticidal efficacy against *A. albopictus* was above 91.1% for 4 weeks after treatment at 4 h PE and was ≥ 97.1% and ≥ 97.8% for 4 weeks after treatment at 24 and 48 h, respectively.

#### Aedes aegypti

The percent insecticidal efficacy against *A. aegypti* on treated dogs was ≥93.8% at 4 h after exposure on Days 1 and 7. At 24 h PE, insecticidal efficacy was ≥98.0% through Day 21 and still 79.8% at Day 28 and 75.7% on Day 35. At 48 h PE, it was ≥ 86.6% for 28 days after treatment and remained at 84.7% on Day 35.

#### Culex pipiens

For *C. pipiens*, no calculation of the insecticidal efficacy was done at 4 h PE because visual observations suggested a too limited efficacy at this early time point. The percent insecticidal efficacy was 77.9% and 79.4% at 24 and 48 h PE, respectively, on Day 1; and 92.1% and 93.8% at 24 and 48 h PE, respectively, on Day 7. Insecticidal efficacy ranged from 93.8% to 30.9%, at 48 h PE during the whole month.

No adverse reactions to the topical treatment were observed in any dog in any of the three studies, including during the 48 h immediately after treatment.

## Discussion

The results of the three studies demonstrate that a single topical treatment of the combination of fipronil and permethrin (Frontline Tri-Act®/Frontect®) provides excellent repellency (inhibition of feeding) against mosquitoes for at least 4 weeks. The product provided immediate effects after administration as demonstrated by the high percent repellency in each study one day after treatment. Repellency on Day 1 was 93.4% against *A. albopictus*, 99.9% against *A. aegypti* and 99.4% against *C. pipiens.* The repellent effects persisted for at least 28 days after treatment with 86.9% repellency against *A. albopictus*, 96.1% repellency against *A. aegypti* and 90.4% repellency against *C. pipiens* on Day 28. Study 2 was extended due to the excellent repellency on Day 28 and repellency against *A. aegypti* remained high at 91.0% on Day 35 after treatment.

The repellency of the fipronil and permethrin combination against *A. aegypti* was similar to, or higher than, previously reported for other commercial permethrin-containing products [9,15,16]. The repellency of a single-active 65% permethrin product ranged from 78.0% to 89.9% until Day 21 and then declined to 61.9% on Day 28; for a combination of permethrin and imidacloprid, repellency ranged from 84.9% to 94.1% through Day 21 and then declined to 50.4% on Day 28; and for a combination of permethrin, dinotefuran and pyriproxyfen repellency ranged from 91.5% to 94.0% through Day 21 and remained at 87.0% on Day 28 [9,15,16]. The excellent duration of effect of the new combination may relate to the product’s formulation, the combination of the effects of permethrin with those of fipronil, or other factors. Fipronil is a broad spectrum insecticide and has been reported to have activity against mosquitoes [21]. Combining two active ingredients that both have activity against mosquitoes may lead to improved activity of the product for a longer duration.

In addition to the strong repellent efficacy, the product demonstrated high insecticidal efficacy on mosquitoes that have been in contact with treated dogs (even those mosquitoes that are repelled). This efficacy was ≥91.1% on *A. albopictus* as early as 4 h after exposure for at least 1 month after application of the product. It was ≥79.8% at 24 h post exposure on *A. aegypti* at Day 28 exposure and, while lower, still provided significant mortality effect on *C. pipiens* with an insecticidal efficacy between 92.1% and 26.9% for a full month (28 days) at 24 h PE. There is no clear explanation of the difference in insecticidal efficacy against *Aedes* spp. and *Culex pipiens*. Interestingly, similar results were observed in preliminary studies performed in another laboratory (data not shown). One hypothesis is that the repellent effect is stronger on *C. pipiens,* preventing them to land on the treated animals as is the case for *Aedes* spp., therefore limiting their contact with the insecticidal molecules.

The mosquito challenge was robust on all exposure days. A high percentage of the mosquitoes fed on the untreated control dogs on each exposure day of each study. The feeding percentages in the current studies are similar to those reported in previous dog studies using *A. aegypti* and *C. pipiens* [9,15,16,21]*.* The feeding rate was lower in Study 1, with a GM of 18.3 to 38.0 (of approximately 75 *A. albopictus* used for each exposure) having fed during the exposure period. The reason for the lower feeding rate of *A. albopictus* as compared to the other species is unknown. While *A. albopictus* will feed on a wide variety of hosts, it has been shown that they tend to have lower feeding rates in general than other mosquito species [22,23] Despite the lower feeding rates, the *A. albopictus* challenge was robust, especially towards the end of the trial, and allowed for a valid test of the repellency of the product.

The product’s repellent effects will help prevent mosquitoes from biting and taking a blood meal from treated dogs, thus protecting dogs from bites that can be painful and cause inflammation and allergic reactions in some animals. In addition, as stated by the WHO, disease prevention through vector control is an important component of disease control and it can reduce disease transmission [24]. Therefore, this repellent efficacy may contribute to the protection of dogs against major diseases transmitted by mosquitoes. In addition, the insecticidal efficacy, illustrating the ability of the product to kill mosquitoes, may be helpful to control the number of mosquitoes in the local vicinity of the treated dog.

The control of *A. albopictus* in particular poses a serious problem [13,25]. This species has become an emerging and important vector in many parts of the world. The biology and behavior of this particular species makes the surveillance and control of it hugely important. The tiger mosquito is very aggressive and feeds during the day, with two main peaks of activity in the morning and early evening [7,23]. It originated in Southeast Asia but has rapidly spread into many other regions of the world. It has been detected much farther north than other similar species (*A. aegypti,* for example), likely due to the fact that it can readily adapt to colder temperatures by becoming dormant in the winter. The species can be difficult to control as it can utilize a large variety of larval breeding sites, from natural sites, such as tree stumps and holes, to artificial containers, such as water storage containers and old pieces of automobiles [14]. It has been shown to be a very competent vector of multiple viruses affecting humans, including dengue virus, chikungunya virus, West Nile virus and others, and has been shown to feed on a variety of hosts. It prefers mammals, but will feed on most vertebrate hosts, including reptiles, birds and amphibians. This not only increases fecundity and survivability of the species, but also increases the risk that it will propagate zoonotic pathogens [14]. This report demonstrates that the combination of permethrin and fipronil and permethrin provides a high level of repellency and insecticidal efficacy against this emerging and difficult to control species in treated dogs for at least 4 weeks.

## Conclusions

The results of the three studies reported here demonstrate that a single topical treatment of a new combination of fipronil and permethrin (Frontline Tri- Act®/Frontect®) provides excellent repellency (inhibition of feeding) and insecticidal efficacy against mosquitoes on dogs for at least 4 weeks. The product may therefore significantly reduce the potential for the transmission of vector-borne diseases through the inhibition of mosquito feeding as well as reducing the discomfort and possible hypersensitivity reactions associated with mosquito bites.
